# Resculpting carbon dots via electrochemical etching

**DOI:** 10.1038/s41598-023-30547-6

**Published:** 2023-03-06

**Authors:** Qingsong Yang, Spyridon Gavalas, Aleksander Ejsmont, Marta J. Krysmann, Jiangtao Guo, Li Li, Xuhong Guo, Antonios Kelarakis

**Affiliations:** 1 State-Key Laboratory of Chemical Engineering, East China University of Science and Technology, Shanghai, China; 2 UCLan Research Centre for Smart Materials, School of Natural Sciences, University of Central Lancashire, Preston, PR12HE UK; 3 Department of Chemical Technology, Faculty of Chemistry, Adam Mickiewicz University in Poznań, Uniwersytetu Poznańskiego 8, 61-614 Poznan, Poland; 4 UCLan Research Centre for Smart Materials, School of Dentistry, University of Central Lancashire, Preston, PR12HE UK; 5 Engineering Research Centre of Materials Chemical Engineering of Xinjiang Bingtuan, Shihezi University, Shihezi, China

**Keywords:** Nanoscale materials, Nanoparticles

## Abstract

Substantial efforts are directed into exploring the structure-properties relationships of photoluminescent Carbon dots (C-dots). This study unravels a resculpting mechanism in C-dots that is triggered by electrochemical etching and proceeds via extensive surface oxidation and carbon–carbon breakage. The process results in the gradual shrinkage of the nanoparticles and can enhance the quantum yield by more than half order of magnitude compared to the untreated analogues.

## Introduction

As one of the most promising types of nano-emitters, Carbon dots (abbreviated as C-dots) exhibit characteristic excitation wavelength-dependent emission and remarkable resistance to photobleaching, showing performance characteristics similar to conventional heavy-metal based quantum dots (QDs)^1–6^. In terms of elemental composition, C-dots mainly consist of C, H, O, N, while a number of studies suggest that the surface functional groups play a prominent role in their dispersibility, colloidal stability, optical properties, toxicity, biocompatibility and cell uptake^7^.

The exact origin of their photoluminescent (PL) behaviour is not fully understood and thus the development of tailor-made C-dots remains an open challenge. Well-defined C-dots can be synthesised *via* thermal treatment of renewable resources^8^ including fruits^9^, grass^10^, wool^11^ or molecular precursors such as urea^12^, ethanolamine^13^, citric acid^14^, folic acid^15^. Pyrolytically derived C-dots are generated in the aqueous phase, in solid-state or in situ within a polymer matrix^16^. Depending on the nature of the starting materials and the synthetic method followed, the graphitization degree of C-dots can vary considerably from essentially amorphous all the way to highly graphitic^17^.

By virtue of their desired characteristics outlined above, C-dots are systematically explored in chemical and biological sensing^18^, bioimaging^19^, nanomedicine^20^, antimicrobial coatings^21^, nano-forensics^22^, fertilisers^23^, energy converters^24^ and electrocatalysis^25^. Suffice it to say that C-dots experience various levels of electrochemical potentials, when used in electrocatalysis, electro-sensing, photovoltaics, batteries and light-emitting diodes. Moreover, electrogeneration of C-dots takes place *via* the exfoliation of electrodes composed of graphene, graphite, carbon fibres, carbon nanotubes^26^, charcoal^27^ (a top-down approach) or *via* electrooxidation/electropolymerization of small molecules precursors such as alcohols^28^, acetonitrile^29^ (a bottom-up strategy). Currently, methods are being pursued to allow rigorous control of C-dots size and their PL emission, thus facilitating further applications^30,31^.

In this work, we disclose an electrochemically triggered mechanism that dramatically modifies the structural characteristics and optical properties of C-dots. The process relies on electrochemical etching and proceeds *via* extensive surface oxidation and breakage of the carbon-carbon bonding. On that basis, the nanoparticle size is gradually diminished, while the quantum yield (QY) is enhanced up to 640%. To the best of our knowledge, this is the first study that contributes solid evidence on the action of this highly effective resculpting mechanism in C-dots that affords possibilities for size tuning and accurate control of their PL emission.

## Results and discussion

The PL spectra of aqueous dispersions of C-dots (SI Fig. 1) display the characteristic λ_ex_ dependent emissive pattern within the range 380 to 500 nm in the sense that the emission wavelength (λ_em_) redshifts upon increasing λ_ex_. This type of emissive mode has been assigned to contributions related to electronic bandgap transitions of conjugated π-domains, surface defect states, edge effects and crosslink enhanced emission, while the presence of molecular chromophores is typically associated with the occurrence of distinct λ_ex_ independent contributions^32–34^.

**Figure 1:**
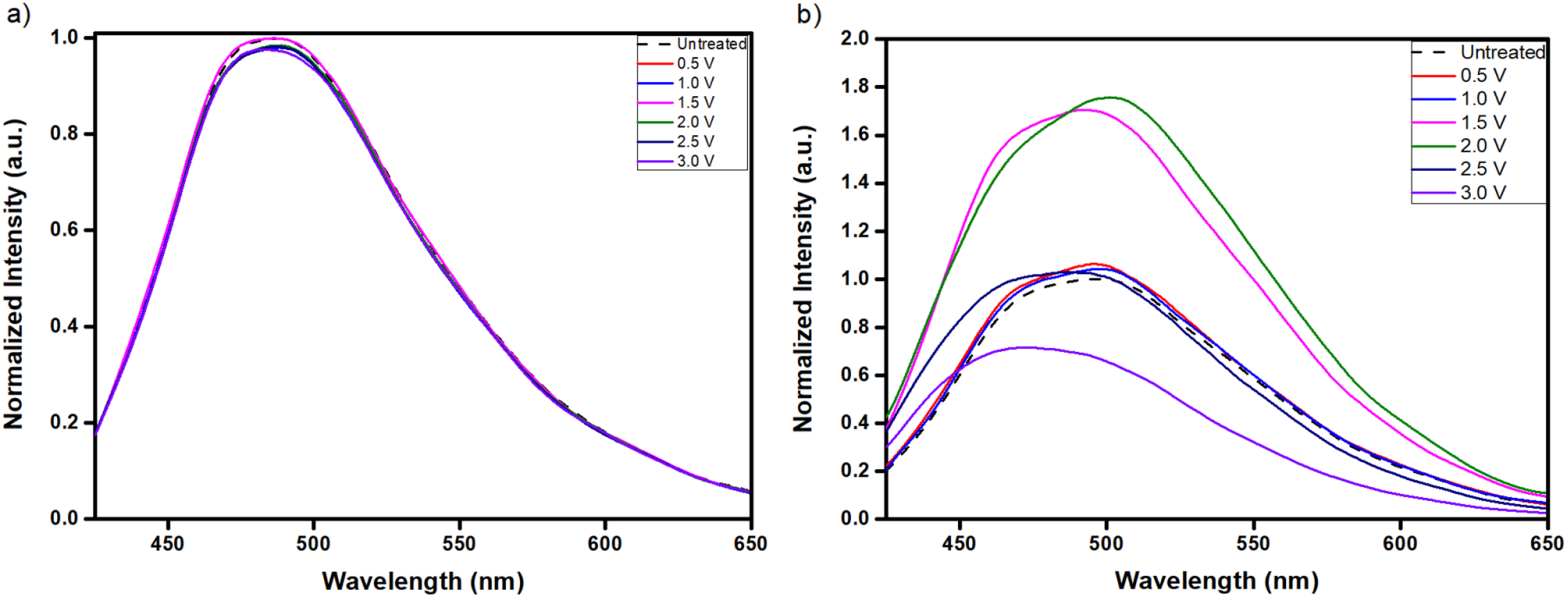
Normalized (against I_max_ of the untreated dispersion) PL spectra (λ_ex_ = 410 nm) of 0.02 mg ml^–1^ C-dots dispersed in 0.15 M aqueous solutions of (**a**) Na_2_CO_3_ and (**b**) KCl that have been subjected to chronoamperometry treatment for 60 s at applied voltage from 0.5 to 3.0 V.

The PL spectra (λ_ex_ = 410 nm) of 0.02 mg ml^−1^ C-dots in 0.15 M Na_2_CO_3_ aqueous electrolyte remain essentially unaltered following chronoamperometry treatments with applied voltage up to 3 V (Fig. 1a), while similar trends were observed for NaHCO_3_ and Na_2_SO_4_ electrolytes. In contrast, when KCl (Fig. 1b) is used as the electrolyte the PL properties of C-dots change significantly and the equilibrium values are reached 48 h after the cessation of the electric field (all data reported hereafter refer to equilibrium values), while similar trends were observed for NaCl and CaCl_2_.

**Figure 2:**
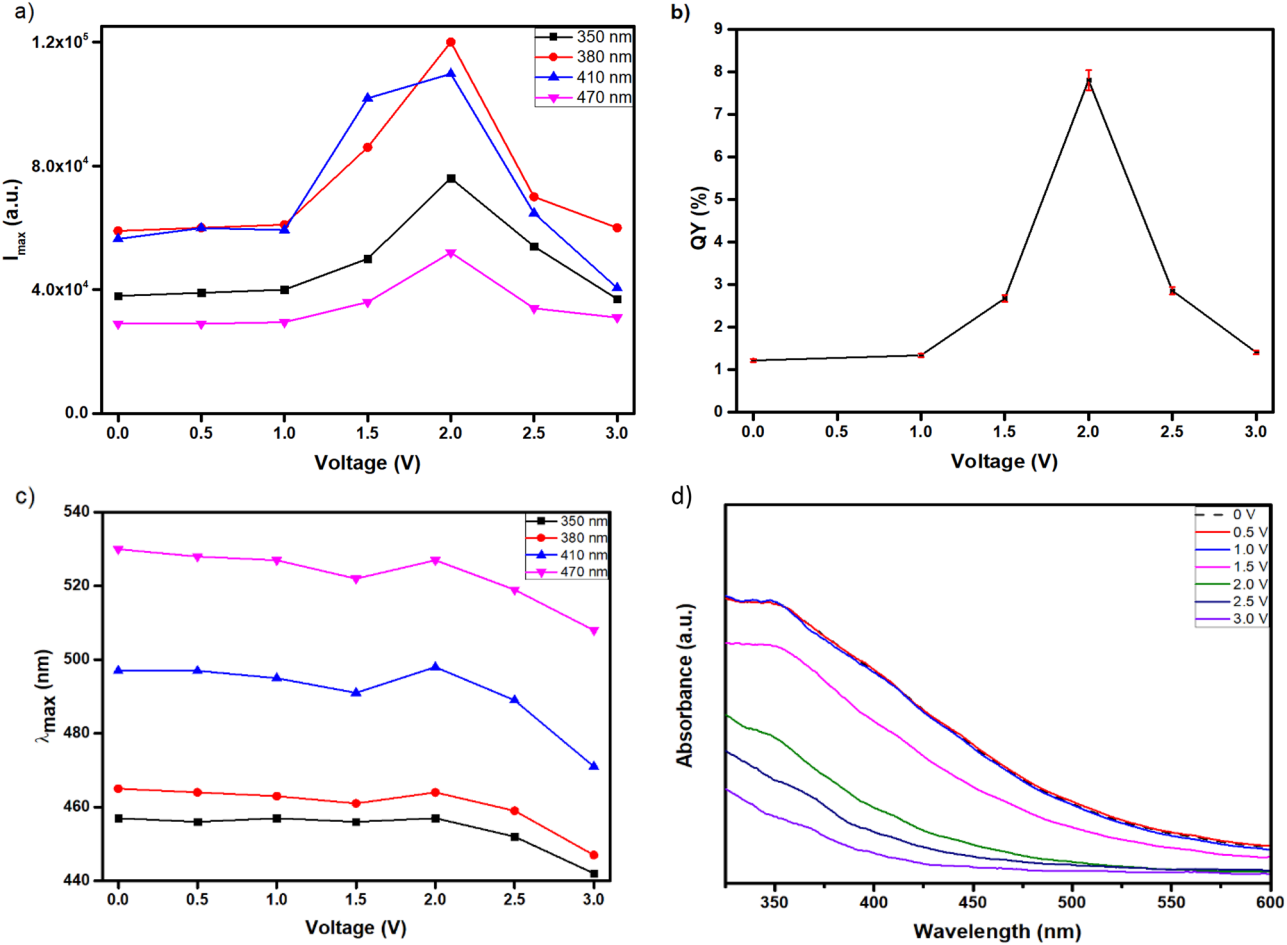
0.02 mg ml^–1^ C-dots dispersion in 0.15 M KCl that has been subjected to chronoamperometry treatments for 60 s within applied voltage from 0.5 to 3.0 V compared to the untreated dispersion. (**a**) Maximum PL intensity (I_max_) at λ_ex_ = 350,380,410, 470 nm, (**b**) QY (λ_ex_ = 365 nm) and (**c**) λ_max_ as a function of the applied voltage, (**d**) UV–vis absorbance spectra.

Αs shown in SI Fig. 2 the maximum emission intensity (I_max_) of electrochemically treated C-dots is seen to increase with KCl concentration up to 0.1 M, exhibits a plateau between 0.1 and 0.2 M and then falls sharply upon further addition of the electrolyte (in all measurements described hereafter the KCl concentration was 0.15 M). Figure 2a suggests that while only minor variations of I_max_ are observed at voltages 0.5 and 1.0 V (λ_ex_ = 410 nm), a substantial increase in I_max_ takes place at 1.5 V and a further enhancement at 2.0 V is followed by a sharp decline at 2.5 V and 3.0 V. Similar trends are observed at λ_ex_ = 350, 380 and 470 nm. In addition, as shown in Fig. 2b the QY (λ_ex_ = 365 nm) of the untreated C-dot dispersions is 1.2% and increases to 1.3%, 2.7%, 7.8%, 2.9% and 1.4% for the samples treated at 1.0, 1.5, 2.0, 2.5, 3.0 V, respectively. Evidently, treatment at 2.0 V gives rise to a redshift consistent with the behaviour expected due to surface oxidation^35^, but the overall trend in Fig. 2c suggests a blue shift, an effect that might be associated with size variations of the carbogenic cores.

**Figure 3:**
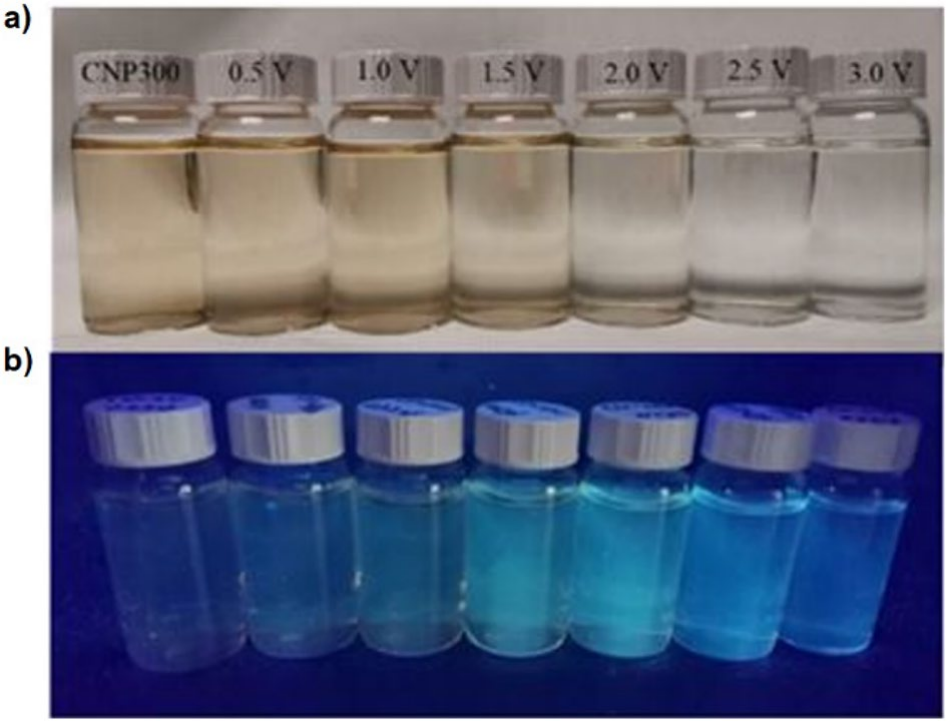
Photos of 0.02 mg ml^–1^ C-dots dispersions in 0.15 M KCl that has been subjected to chronoamperometry treatments for 60 s at applied voltages from 0.5 to 3.0 V compared to the untreated sample under (**a**) daylight and (**b**) UV-radiation.

At the same time, the UV-vis absorbance of the samples treated at 0.5 V and 1.0 V remains close to that shown by the untreated dispersion, however it drops progressively as the applied voltage is increased at 1.5, 2.0, 2.5 and 3.0 V (Fig. 2d). Figure 3a and b compare the untreated dispersion with photos of the C-dots dispersions that have been subjected to chronoamperometry treatments at voltages 0.5, 1.0, 1.5, 2.0, 2.5, 3.0 V under daylight and UV radiation, respectively. Clearly, the samples treated at 1.5 and 2.0 V appear to have the highest levels of fluorescence, in line with the behaviour shown in Fig. 2a and b.

We note that during the chronoamperometry treatment at 3.0 V for 60 s the temperature of the C-dot dispersion was raised from 26.4 to 27.9 (± 0.2) ^o^C and the pH was increased from 5.7 to 6.4 (± 0.1), while even more limited changes were recorded during treatments at lower voltages. Given that the PL spectra of C-dots remain essentially unaffected when the temperature and/or the pH are adjusted within this narrow range, the effect of those parameters on the pronounced changes in PL intensity reported here seems to be marginal.

**Figure 4:**
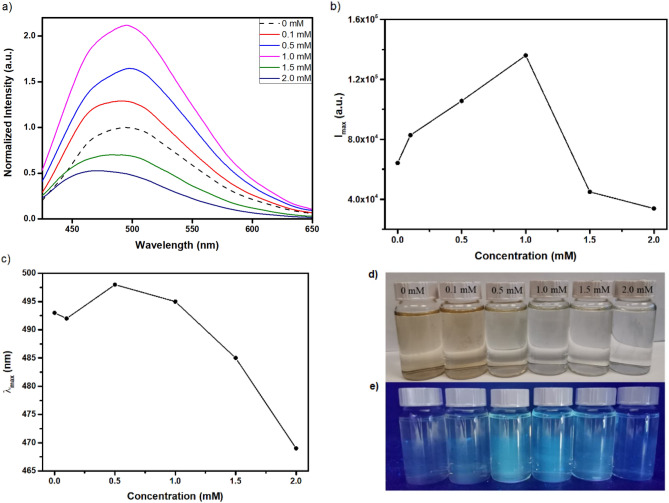
0.02 mg ml^–1^ C-dots dispersed in aqueous solution of NaClO with salt concentrations 0.1, 0.5, 1.0, 1.5 and 2.0 mM, compared to the untreated sample. (**a**) PL spectra (λ_ex_ = 410 nm), (**b**) the corresponding maximum PL intensity (I_max_) and (**c**) λ_max_ as a function of the electrolyte concentration, photos of C-dots dispersions under daylight (**d**) and (**e**) UV radiation. All data have been collected 48 h after the dispersion of C-dots in the aqueous solution of NaClO.

We hypothesize that the dramatic modifications in PL properties of C-dots are directly related to the electro-generation of hypochlorite ions (ClO^–^)^36^, a well-explored effect that is employed in water purification^37^. Iodometric titration using sodium thiosulfate indicated that the KCl solution treated at 3 V for 60 s contained 3.5 mM hypochlorite ions. To further explore this effect, Fig. 4a displays the PL spectra of C-dots recorded 48 h following their dispersion in NaClO solutions with varying electrolyte concentrations. As plotted in Fig. 4b, I_max_ monotonically increases with NaClO concentration up to 1 mM, but then drops abruptly upon further increase of the electrolyte concentration. At the same time, the wavelength of the I_max_ (λ_max_) is seen at 493 nm in water, shifts at 498, 485 and 469 in the presence of 0.5 M, 1.5 M and 2.0 M NaClO (Fig. 4c). Τrends observed in Fig. 4b and c show similarities with those observed in Fig. 2b and c, respectively. Note that data plotted in Fig. 4 are not associated with any type of voltametric process and are attributed exclusively to the oxidative nature of the NaClO. The gradual decolouration of C-dots dispersions in the presence of increasing levels of NaClO (Fig. 4d) points to major structural changes induced by the electrolyte. Interestingly, the PL properties of NaClO preoxidised C-dots, undergo only minor changes when are subsequently subjected to chronoamperometry treatments (SI Fig. 3).

It has been supported that the strong nonspecific oxidant hypochlorite (ClO^–^) can facilitate the cleavage of carbon-carbon double bonds (C=C)^38^ and the imine groups (C=N)^39^, while it can also attack ether bonds and the hydroxyl groups. The degradation of GO from hypochlorite under UV light gives rise to the release of graphene dots^39^ given that epoxy and alkoxy units on the basal plane of GO are attacked by NaClO and the skeletal carbon bonds are broken down, while peripheral groups are oxidized into COOH^40^. Similarly, electrochemical exfoliation of graphite rods immersed in KCl aqueous electrolyte under applied voltages 9–30 V leads to the generation of C-dots with a diameter close to 2 nm^41^. In the light of the evidence reported here, it seems reasonable to assume that this type of previously reported behaviour might be also associated with the occurrence of electrogenerated hypochlorite ions.

A recent study suggested that the oxidative degradation induced by hypochlorite is faster for graphene oxide compared to oxidised carbon nano-horn (CNHs) and multi-walled carbon nanotube (MWNT)^42^. The degradation kinetics follows the order: single-walled carbon nanotube (SWNTs) ≥ CNHs > thinner MWNTs > thicker MWNTs^43^. Another study suggests that the hypochlorite-degraded graphene oxide shows lower levels of toxicity on *Caenorhabditis elegans* due to the enhanced population of surface oxygen groups^44^.

Coming back to the chronoamperometry experiments employed in this study, we note that the FTIR spectrum of the untreated C-dots (lower black line in S.I. Fig. 4) shows peaks centred at 935 and 850 cm^–1^, both corresponding to the bending vibration of the C=C–H bond of the sp^2^ carbon, but those peaks cannot be discerned in the treated samples (upper red line in Fig. 3b). Similarly, the peaks at 1185, 1653 and 1691 cm^–1^ corresponding to C–N, C=O and C=O/C=N, respectively appear weaker on the electrochemically etched C-dots.

**Figure 5:**
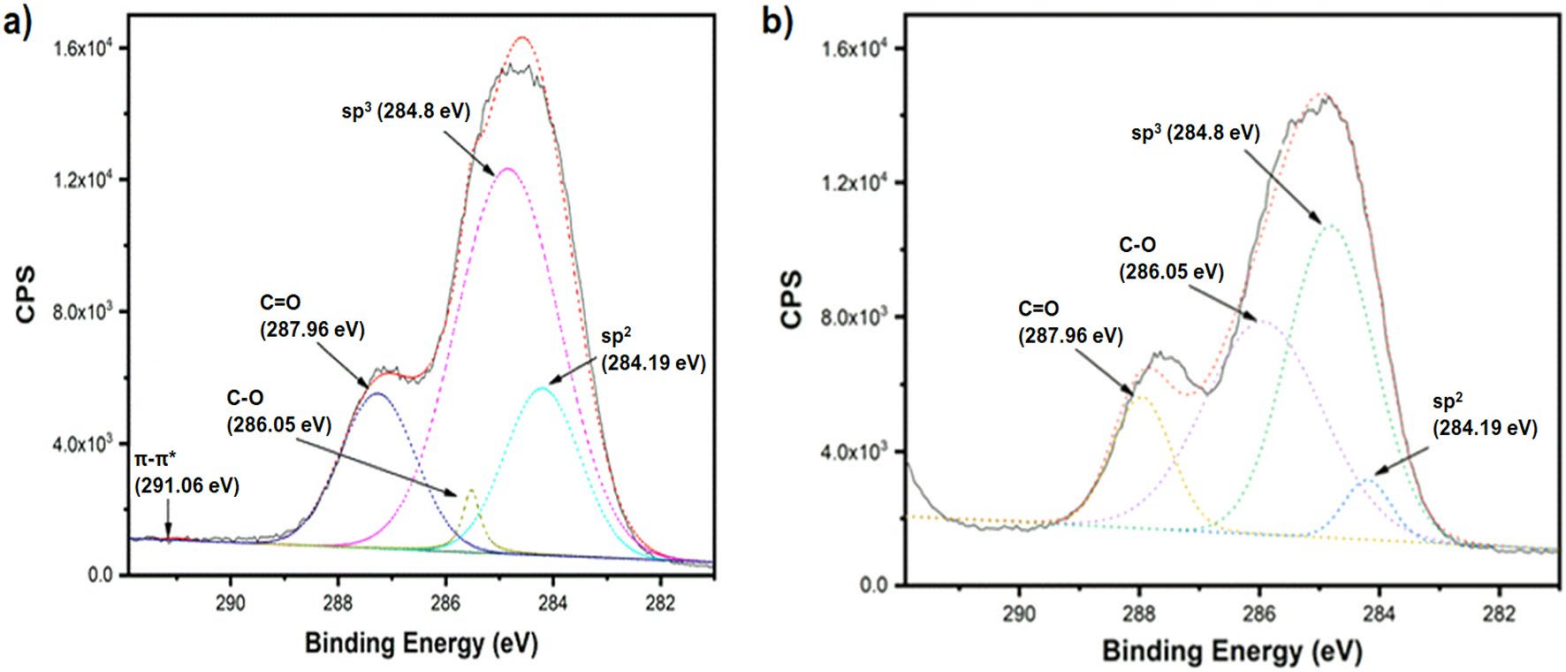
C1s XPS spectra of C-dots prior (**a**) and after (**b**) chronoamperometry treatment at 2.0 V for 60 s. Solid lines refer to the recorded data, while the dashed lines refer to the fitted curves.

Moreover, XPS analysis reveals that the C1s XPS spectrum of the untreated C-dots (Fig. 5a) is dominated by 60.6% sp^3^, 19.1% sp^2^ carbon (accompanied by a minor p–p* satellite peak), 17.8% C=O and 2.4% C–O. Following the chronoamperometry treatment (2.0 V for 60 s), XPS analysis (Fig. 5b) indicates reduced percentages of 42.4% sp^3^, 4.9% sp^2^, 13.1% C=O, while the contribution of C–O increases drastically to 39.6%. Data derived from C1s XPS analysis are summarised in S.I. Tables 1 and 2. FTIR and XPS data considered together, suggest that the application of the electric field results in extensive surface oxidation and pronounced breakage of the carbon-carbon bonding.

**Figure 6:**
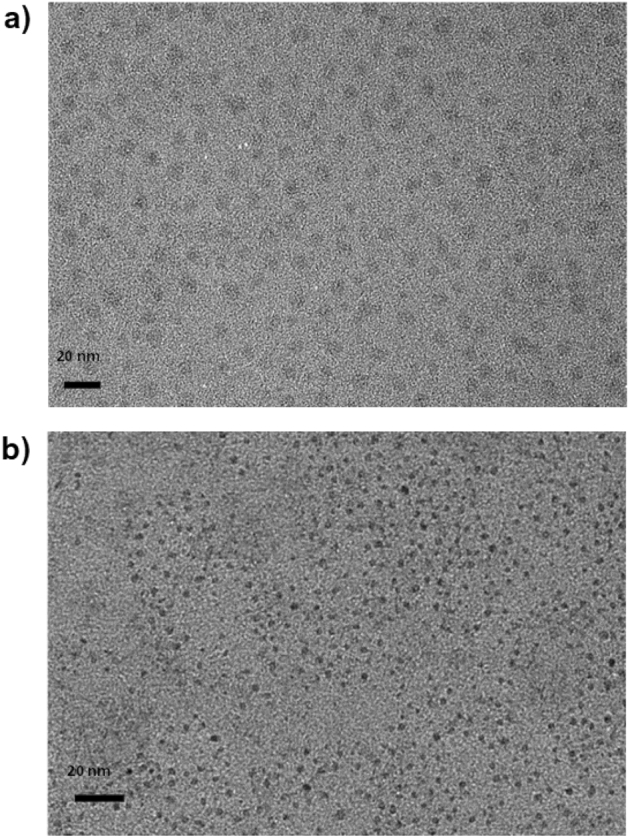
TEM images of C-dots prior (**a**) and after (**b**) been subjected at chronoamperometry treatment at 2.0 V for 60 s.

TEM images indicate that the average diameter of untreated C-dots is close to 12 nm (Fig. 6a), while after the application of the electrochemical field (2.0 V for 60 s) the nanoparticles maintain their spherical symmetry, but their diameter falls close to 3 nm (Fig. 6b). This significant shrinkage of C-dots induced by the electric field is consistent with the extensive surface oxidation and disintegration of the skeleton carbon described above. In a further experiment, the chronoamperometry process was repeated five consecutive times (2.0 V, 60 s each) and the resulting dispersion showed near-zero PL emission, while no structural features could be detected by TEM, pointing to the complete decomposition of the suspended nanoparticles.

Of relevance here is the photodegradation mechanism identified for certain types of C-dots. In particular, the photo-degradation of C-dots electrolytically derived from vitamin C into CO_2_, CO and H_2_O is completed after twenty days of continuous irradiation with visible light^45^. In another study, the photodegradation of C-dots derived *via* microwave-assisted pyrolysis of PEG/glucose has been attributed to the occurrence of photogenerated hydroxyl and alkyl radicals in a manner that critically depends upon the light intensity and wavelength as well as pH and temperature^46^.

For reference, it is noted that the PL emission of reduced C-dots is quenched and red-shifted by oxidants such as KMnO_4_, KIO_4_ and K_2_Cr_2_O_7_ that selectively oxidize hydroxyl groups, but the process is fully reversible and does not involve any change in the size of nanoparticles^35^. Earlier studies indicated that C-dots derived in a similar manner as those described herein (via pyrolysis of EA and CA at 300 °C) when subjected to cyclic voltammetry treatment within the potential window − 2.0 to 0.5 V (i.e. a very different treatment compared to the chronoamperometry treatment of 1 min at voltages up to 3 V followed in the present study) showed a 2% PL quenching and their size remain essentially unchanged^47^. For comparison, we note that the C-dots considered in the present study showed a 2-fold enhancement in their PL intensity when subjected to 15 sweeps of cyclic voltammetry within the potential window − 2.5 to 2.5V, using KCl as the electrolyte.

## Conclusion

The present study reveals for the first time a previously unexplored restructuring mechanism of C-dots induced by electrochemical etching that manifests itself *via* the gradual shrinkage of the nanoemitters and the drastic changes in their optical properties. The QY of the electrochemically etched C-dots reflects the interplay between the number and the size of the emissive nanoparticles, their surface oxidation states and the conjugation level of the carbon skeleton. Our study sheds some light on the role of the electrogenerated hypochlorite anions in attacking the surface of nanoparticles, progressively leading to complete disintegration within a period of few minutes.

## Materials and methods

### Materials

Citric acid (CA), nitric acid (HNO_3_), potassium chloride (KCl), sodium chloride (NaCl), calcium chloride CaCl_2_, potassium iodide (KI), sodium sulfate (Na_2_SO_4_), sodium carbonate (Na_2_CO_3_), sodium hydrogen carbonate (NaHCO_3_), sodium hypochlorite (NaClO), sodium thiosulfate (Na_2_S_2_O_3_), starch, anthracene were purchased by Sigma-Aldrich, while ethanolamine (EA) was purchased by Alfa Aesar.

### Synthesis of C-dots

C-dots were synthesized by means of pyrolysis of a mixture comprising citric acid (CA) and ethanolamine (EA) according to a protocol described elsewhere^32^. In a typical run, 30.00 g CA and 28.61 g EA were mixed in a round bottom flask and heated at 180 ℃ for 30 min under reflux in air. Then the condenser was removed, and the temperature was increased to 230 ℃. The reaction was carried out for 30 min and the product was transferred to a crucible and the product was further pyrolyzed in the oven at 300 ℃ for 1 h. Subsequently, the product was treated with HNO_3_ (400 ml, 1.5 M) at 100 °C for 16 h. The oxidized product was purified by dialysis using SnakeSkin Pleated Dialysis Tubing membrane (with a molecular weight cut-off of 3500 Da) and freeze-dried.

### Electrochemical treatment

Chronoamperometry treatments were carried out on a Gamry Interface 1000 potentiostat at room temperature using a three-electrode system consisted of an Ag/AgCl electrode as the reference electrode, and two platinum electrodes as the working electrode and counter electrode, respectively. Unless otherwise specified, the C-dots were dispersed in 0.15 M KCl solution, the magnitude of the applied voltage varied from 0.5 to 3.0 V (with 0.5 V increment). In all tests, the duration of the treatment was kept constant at 60 s.

### Chemical treatment

10 ml of 0.04 mg ml^–1^ aqueous dispersion of C-dots was added under stirring into beakers containing 10 ml of NaClO with concentrations 0.1, 0.5, 1.0, 1.5, 2.0 mM. All mixtures were left at room temperature for 48 h before their PL spectra were recorded.

### Iodometric titration of hypochlorite ions

20 ml H_2_SO_4_ (0.1M), and 10 ml KI (0.5M) along with 20 ml of the KCl solution (0.15M) that has been subjected to amperometry treatment (3V, 60 s) was added in a conical flask. The mixture was titrated with Na_2_S_2_O_3_ solution (0.2 M). The disappearance of the blue colored complexes formed after the addition of starch was used to determine the end point of the titration.

### Ultraviolet–visible (UV–Vis) spectra

Aqueous dispersion were inserted in Hellma Analytics quartz cuvette (1.0 cm pathlength) and their specta were recorded at room temperature using the UV-3600 spectrophotometer (Shimadzu).

### Photoluminescence spectra

Photoluminescence spectra of aqueous dispersions of C-dots were recorded using a Horiba Fluoromax spectrofluorometer at excitation wavelengths (λ_ex_) between 320 and 500 nm. The QY was determined *via* the equation:

$$\mathrm{QY}={\mathrm{QY}}_{\mathrm{R}}\times \left(\frac{\mathrm{I}}{{\mathrm{I}}_{\mathrm{R}}}\right)\times \left(\frac{{\mathrm{A}}_{\mathrm{R}}}{A}\right)\times \left(\frac{{\upeta }^{2}}{{\upeta }_{\mathrm{R}}^{2}}\right)$$

where, I, A, and η denote the integrated fluorescence intensity, absorbance, and the refractive index, respectively. The subscript R indicates the reference dye anthracene that was dissolved in ethanol giving QY_R_ = 0.27 at λ_ex_ = 365 nm. The error bars have been calculated based on a series of three independently repeated experiments.

### X-ray photoelectron spectroscopy (XPS)

The XPS spectra of C-dots were recorded using ESCALAB 250Xi spectrometer (Thermo Fisher) equipped with a monochromatic Al Kα Xray radiation source. The data were fitted using the Thermo Avantage software.

### Fourier transform infrared (FTIR)

FTIR spectra were recorded by Nicolet IS5 spectrometer within the range of 4000–500 cm^–1^ and the dried samples were scanned 128 times at a resolution of 2 cm^–1^.

### Transmission electron microscopy (TEM)

TEM images were obtained using a Tecnai F20 microscope operated at 200 kV. Digital imaging was accomplished *via* an eagle camera and TIA software. The samples recovered after chronoamperometry were subjected to dialysis against water to remove the electrolytes. A drop of C-dots in ethanol was deposited on the carbon-coated copper grid and the solvent was left to evaporate at room temperature. The diameter of C-dots reported here represent the average of 50 readings using a suitable software.

## Supplementary Information

Supplementary Information.

## Data Availability

The datasets used and/or analysed during the current study are available from the corresponding author on reasonable request.
