# Supplementary material for: Resculpting carbon dots via electrochemical etching
Source: Sci Rep. 2023 Mar 6;13:3710. doi: 10.1038/s41598-023-30547-6 (PMC9988976; doi:10.1038/s41598-023-30547-6)
Supplement: Supplementary file 1 — Supplementary Information. [file 41598_2023_30547_MOESM1_ESM.docx]

Electronic Supplementary Information

**Resculpting Carbon dots via electrochemical etching**

Qingsong Yang^1,2^, Spyridon Gavalas^2^, Aleksander Ejsmont^2,3^, Marta J. Krysmann^4^, Jiangtao Guo^1^**,** Li Li^1^, Xuhong Guo^1,5^*, Antonios Kelarakis^2^*

^1^State-Key Laboratory of Chemical Engineering, East China University of Science and Technology, Shanghai, China,

^2^UCLan Research Centre for Smart Materials, School of Natural Sciences, University of Central Lancashire, Preston PR12HE, UK

^3^Adam Mickiewicz University in Poznań, Faculty of Chemistry, Department of Chemical Technology, Uniwersytetu Poznańskiego 8, 61-614 Poznań, Poland

^4^UCLan Research Centre for Smart Materials, School of Dentistry, University of Central Lancashire, Preston PR12HE, UK

^5^Engineering Research Centre of Materials Chemical Engineering of Xinjiang Bingtuan, Shihezi, University, Shihezi, China


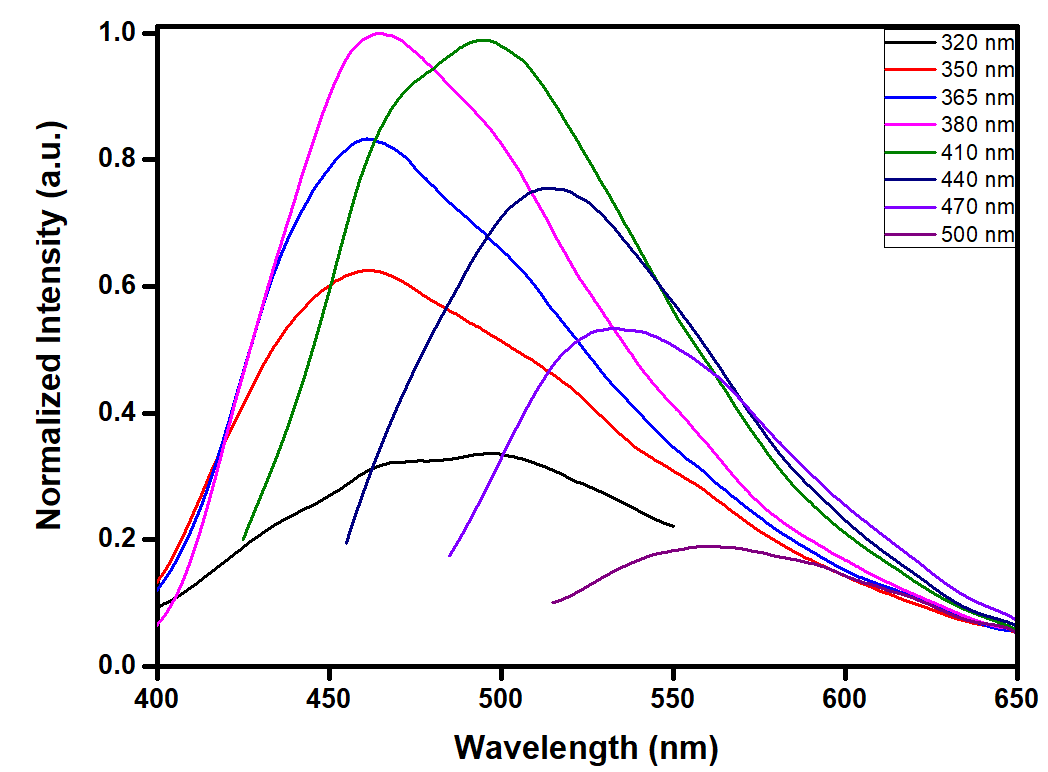


SI Figure 1. Normalized (with respect to the I_max_ at λ_ex_=380 nm) PL spectra of 0.01 mg mL^–1^ aqueous dispersions of C-dots. The excitation wavelength (λ_ex_) was varied from 320 to 500 nm as indicated.


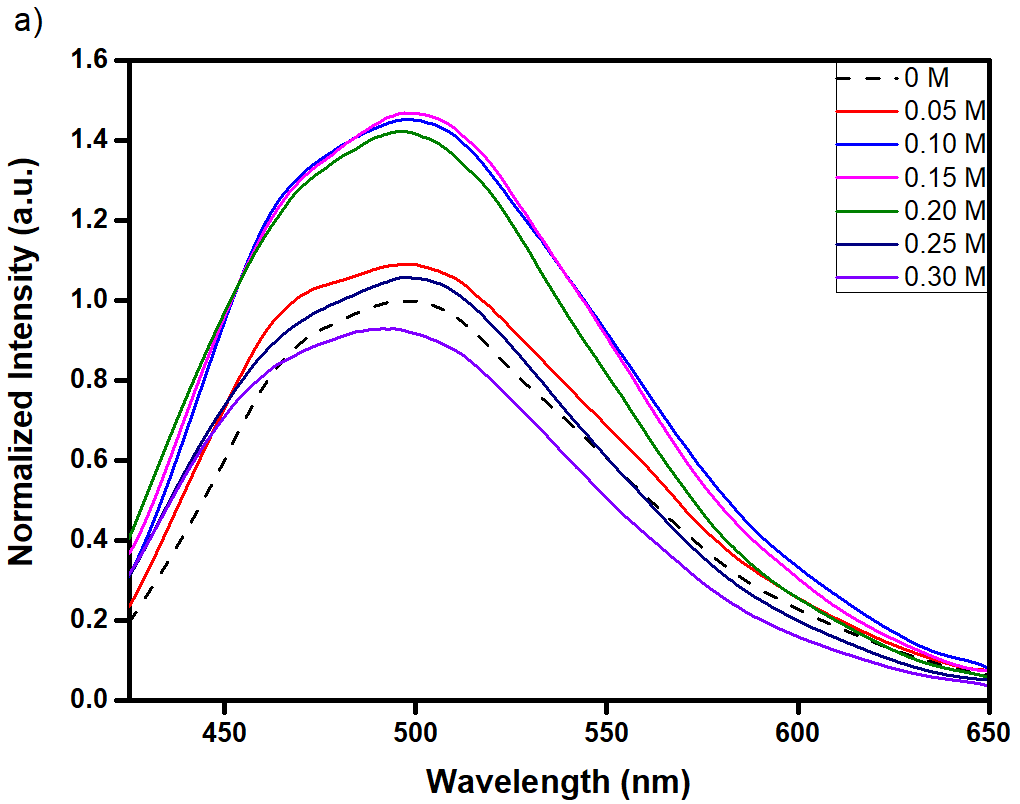

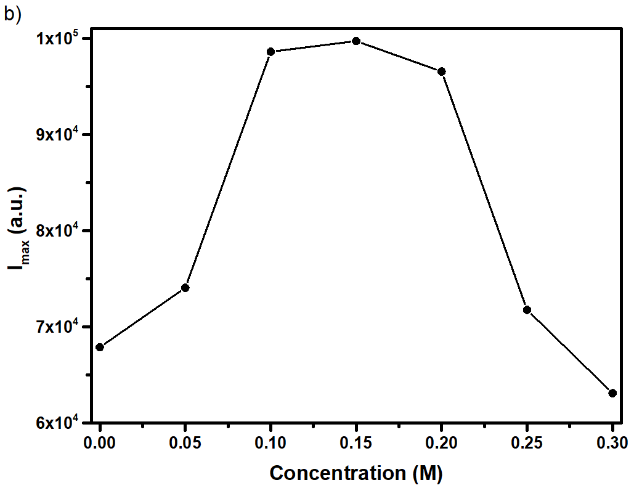


SI Figure 2. 0.02 mg ml^–1^ C-dots dispersions that have been subjected to chronoamperometry treatments for 60 s at 2.0 V in the presence of 0.05, 0.10, 0.15, 0.20, 0.25, 0.30 M KCl. (a) PL spectra (λ_ex_ = 410 nm) and (b) the corresponding maximum PL intensity (I_max_) as a function of KCl concentration.


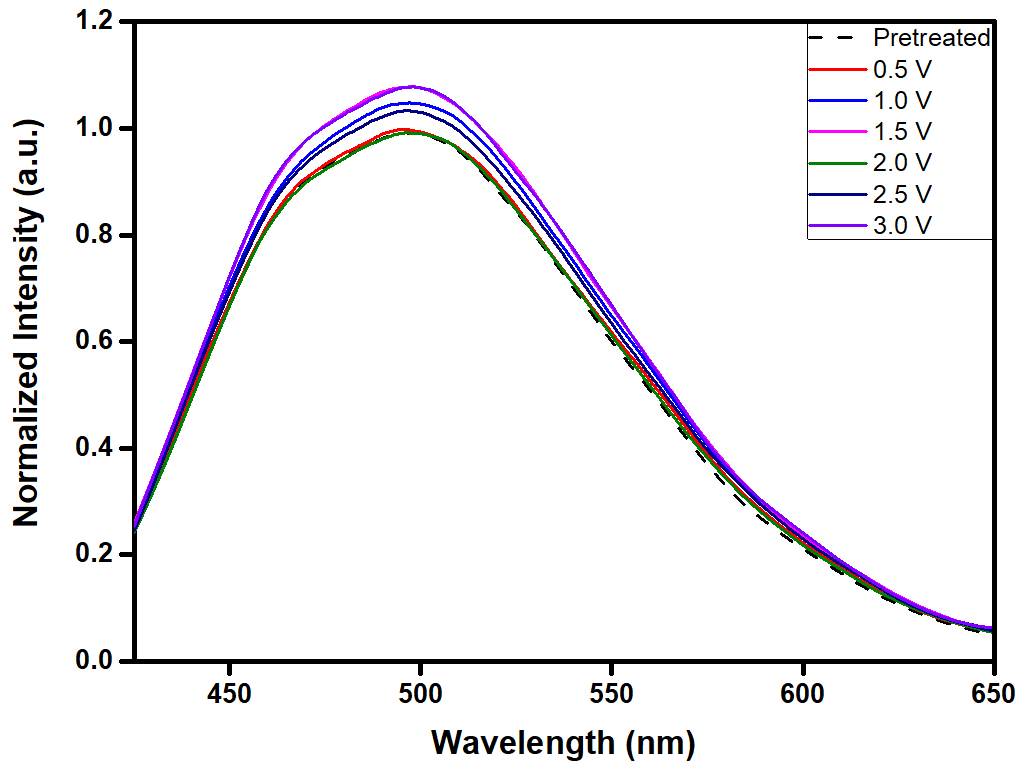


**SI Figure 3**. PL spectra (λ_ex_ = 410 nm) of 0.02 mg ml^–1^ C-dots pretreated with 1mM NaClO solution for 48 h and then subjected to chronoamperometry treatments for 60s at the voltages indicated.


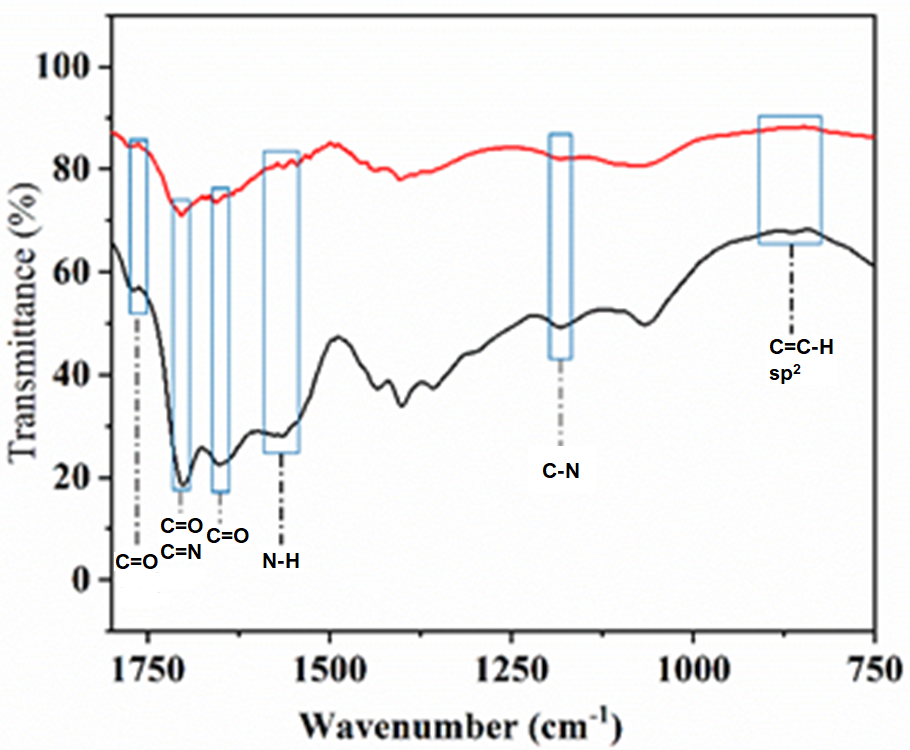


**SI Figure 4.** FTIR spectra of C-dots prior (lower black line) and after (upper red line) the chronoamperometry treatment at 2.0 V for 60 s.

**S.I. Table 1.** Analysis of the C1s XPS spectrum of untreated C-dots.

| Chemical Bind | Combined energy peak position (eV) | Half peak width (eV) | Peak area % |
| --- | --- | --- | --- |
| sp^2^ | 284.19 | 1.63 | 19.1 |
| sp^3^ | 284.8 | 2.26 | 60.6 |
| C–O | 286.05 | 0.50 | 2.4 |
| C=O | 287.96 | 1.65 | 17.8 |
| π–π* | 291.05 | 0.50 | 0.1 |

**S.I. Table 2.** Analysis of the C1s XPS spectrum of C-dots that have been subjected to chronoamperometry treatment a 2.0 V for 60 s.

| Chemical Bind | Combined energy peak position (eV) | Half peak width (eV) | Peak area % |
| --- | --- | --- | --- |
| sp2 | 284.2 | 0.96 | 4.9 |
| sp3 | 284.81 | 1.7 | 42.4 |
| C–O | 285.94 | 2.29 | 39.6 |
| C=O | 287.97 | 1.17 | 13.1 |
